# Interrogating the role of the milk microbiome in mastitis in the multi-omics era

**DOI:** 10.3389/fmicb.2023.1105675

**Published:** 2023-02-02

**Authors:** Sneha P. Couvillion, Katie E. Mostoller, Janet E. Williams, Ryan M. Pace, Izabel L. Stohel, Haley K. Peterson, Carrie D. Nicora, Ernesto S. Nakayasu, Bobbie-Jo M. Webb-Robertson, Mark A. McGuire, Michelle K. McGuire, Thomas O. Metz

**Affiliations:** ^1^Pacific Northwest National Laboratory, Earth and Biological Sciences Directorate, Richland, WA, United States; ^2^Department of Animal, Veterinary, and Food Sciences, University of Idaho, Moscow, ID, United States; ^3^Margaret Ritchie School of Family and Consumer Sciences, University of Idaho, Moscow, ID, United States

**Keywords:** milk, multi-omics, mastitis, metagenomics, metatranscriptomics, metaproteomics, metabolomics, lipidomics

## Abstract

There is growing interest in a functional understanding of milk-associated microbiota as there is ample evidence that host-associated microbial communities play an active role in host health and phenotype. Mastitis, characterized by painful inflammation of the mammary gland, is prevalent among lactating humans and agricultural animals and is associated with significant clinical and economic consequences. The etiology of mastitis is complex and polymicrobial and correlative studies have indicated alterations in milk microbial community composition. Recent evidence is beginning to suggest that a causal relationship may exist between the milk microbiota and host phenotype in mastitis. Multi-omic approaches can be leveraged to gain a mechanistic, molecular level understanding of how the milk microbiome might modulate host physiology, thereby informing strategies to prevent and ameliorate mastitis. In this paper, we review existing studies that have utilized omics approaches to investigate the role of the milk microbiome in mastitis. We also summarize the strengths and challenges associated with the different omics techniques including metagenomics, metatranscriptomics, metaproteomics, metabolomics and lipidomics and provide perspective on the integration of multiple omics technologies for a better functional understanding of the milk microbiome.

## Introduction

The human body is host to trillions of bacterial cells that operate in connected and complex networks to potentially affect host health (Sharma and Gilbert, 2018). As the field of microbiome research advances through the refinement of analytical tools and methodologies, unexpected sites and secretions with diverse microbial communities have been identified including human milk. Except for specific cases of maternal systemic viral infection (e.g., HIV, CMV; Jones, 2001) or local inflammation (e.g., mastitis), milk was historically considered to be a sterile fluid unless contaminated *via* external sources (Fernandez et al., 2013). However, over the past two decades, numerous studies have confirmed the presence of bacteria in milk *via* culture-dependent (Martín et al., 2003; Perez et al., 2007; Delgado et al., 2009) and culture-independent methods (Collado et al., 2009; Hunt et al., 2011; Jiménez et al., 2015; Williams et al., 2017a,b; Moossavi et al., 2019b). This has propelled interest in this ecological niche for its potential to impact both maternal and offspring health (Fernandez et al., 2013; Oikonomou et al., 2020). Beyond that, alterations in the microbiota of milk produced by dairy animals are thought to influence milk production, processing, and spoilage, as well as overall consumer health - intriguing nutritionists, food scientists, and agriculturists alike (Quigley et al., 2013; Parente et al., 2020). The milk microbiome, however, is relatively understudied compared to other human microbiomes such as those of feces [as a proxy for the lower gastrointestinal (GI) tract (Shreiner et al., 2015)], skin (Grice and Segre, 2011), and vagina (France et al., 2022), which have been included in numerous investigations and large collaborative efforts like the Human Microbiome Project (Integrative, 2019). The growing understanding of the impact of host-associated microbial communities on host phenotype and health has increased interest in investigating the milk microbiome. This area of research represents an exciting frontier in maternal and infant health.

The microbial communities in milk are likely influenced by a complex interplay of exchanges among the mother, infant, and environment. Through metagenomics, metatranscriptomics, metaproteomics, and metabolomics studies, it is now possible to elucidate the microbes that constitute the microbial community, their functional potential, metabolic activity, and the products they participate in and produce (respectively) in close interaction with the host. Although the use of high-throughput analytical technologies to characterize the bacterial members of the milk microbiome has primarily focused on taxonomic profiling *via* 16S rRNA gene analysis and whole genome shotgun metagenomic sequencing (Zimmermann and Curtis, 2020). Functional profiling represents an intriguing future direction as demonstrated in more thoroughly studied microbial communities, such as those in the GI tract (Heintz-Buschart and Wilmes, 2018). Shifting the focus of milk microbiome investigations to gain functional understanding could lead to deeper insights into the causal relationships between composition of microbial communities and variation in maternal and infant health.

Omics technologies have been described as “high-throughput biochemical assays that measure comprehensively and simultaneously molecules of the same type from a biological sample” (Conesa and Beck, 2019). In this review, we aim to draw attention to the limited number of existing studies that have used omics approaches to investigate the milk microbiome, especially in the context of mastitis, which is inflammation of the mammary gland that can pose serious clinical and economic consequences for both dairy cattle and women. Clinically, mastitis is characterized by inflammation, often accompanied by breast pain and irritation, complicating the ability to breastfeed. Nearly 10 % of U.S. breastfeeding mothers experience clinical mastitis (Spencer, 2008), and it is of interest to uncover the nutritional and microbial impacts, as it is standard practice to encourage mothers to continue breastfeeding during a mastitis episode (Spencer, 2008). Sub-clinical mastitis in dairy cattle, is typically identified by high somatic cell count (SCC), a marker of mammary inflammation measurable in milk. As there may be no visible signs such as abnormal milk or soreness of the udder, sub-clinical mastitis can escape detection and result in decreased milk production, and poor animal health (Schukken et al., 2003). In humans, sodium/potassium the (Na+/K+) ratio in milk has been used as an indicator of sub-clinical mastitis (Willumsen et al., 2003; Pace et al., 2022). Clinical mastitis in dairy cattle is typically noted by observation of abnormalities in the milk or udder and a positive reaction in the California Mastitis Test. A variety of opportunistic pathogens that are members of the normal host microbiota (e.g., staphylococci and streptococci) have been associated with mastitis, which is characterized by a complex etiology that is likely polymicrobial and variable (Boix-Amorós et al., 2020). There is increasing interest in obtaining a mechanistic understanding of dysbiosis in the milk microbiome in the development and progression of mastitis (Hoque et al., 2020). A dysfunctional GI microbiota has been shown to lead to mastitis following fecal transplantations in animal models (Ma et al., 2018; Hoque et al., 2022), and probiotics that restore intestinal microbiota are effective in preventing and treating mastitis. These findings suggest that a holistic interrogation of host-associated microbial communities and their molecular crosstalk with the host is critical to uncover the microbial etiology of mastitis and develop effective prevention and treatment strategies. Here, we use the term “milk-omics” to describe the holistic and comprehensive characterization of both microbes and the complex composition (e.g., proteins, lipids, carbohydrates, metabolites, and myriad other substances) found in milk.

**Figure 1:**
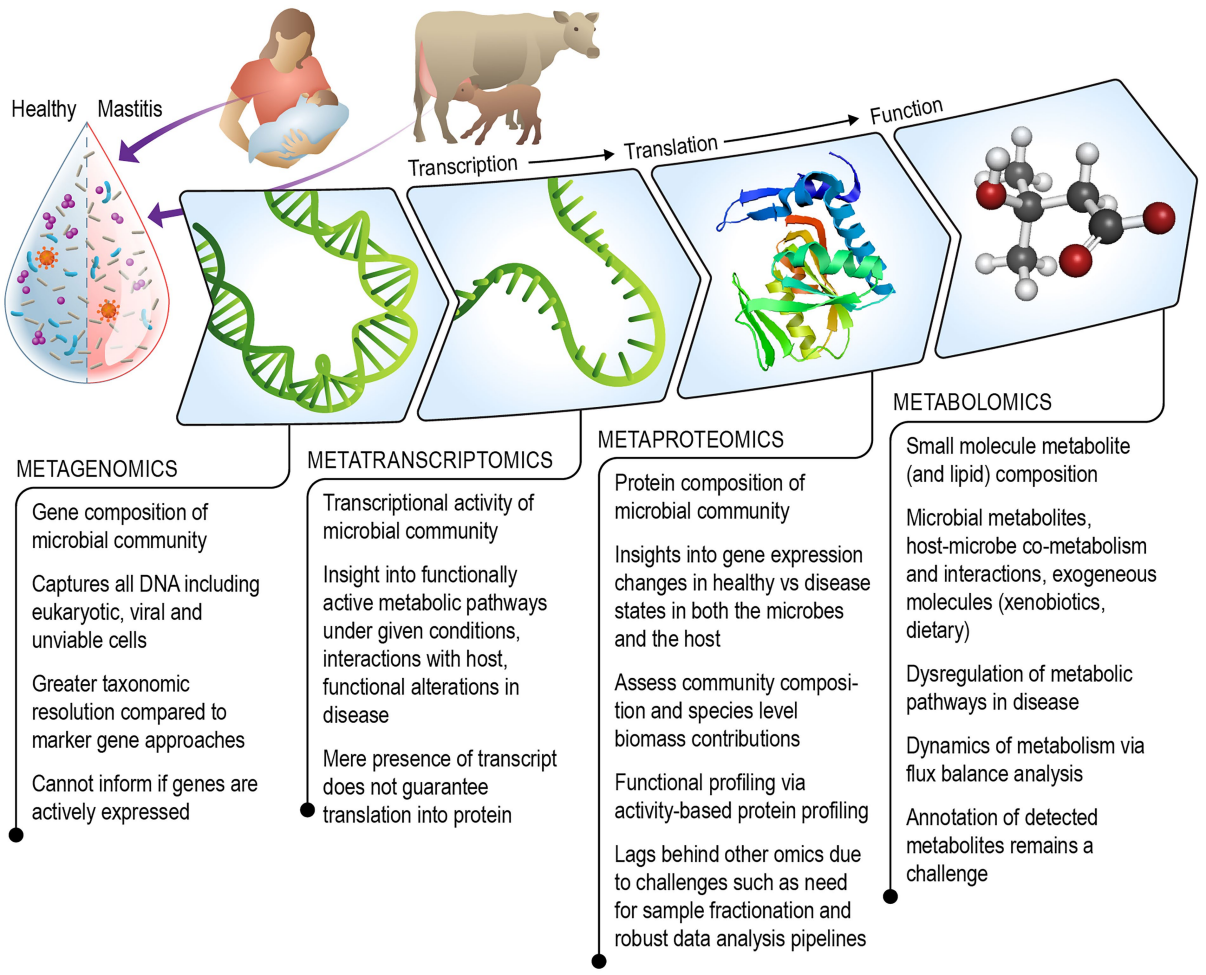
Schematic illustrating the combined multi-omics technologies that can be leveraged to gain a mechanistic understanding of the role of the milk microbiome in the onset and progression of mastitis in humans and agricultural animals.

We review existing literature and discuss the strengths and challenges of each omics technique including metagenomics, metatranscriptomics, metaproteomics, metabolomics (which includes lipidomics), for understanding the role of the milk microbiome in mastitis (Figure 1). Additionally, we consider ways in which the field of milk microbiome research might benefit from multi-omics approaches that have been successfully used to gain systems-level understanding of other microbial communities.

## Origin and importance of milk microbiota

The microbial communities of milk are hypothesized to arise through multiple routes, including exchange from infant to mother (Fernandez et al., 2013). It has been proposed that bacteria present in the infant’s oral cavity help to shape milk microbial communities *via* retrograde flow of bacteria from the infant’s mouth to the breast and mammary ducts. This hypothesis is supported, in part, by the finding that *Streptococcus* species are dominant in both the infant oral cavity and human milk (Cephas et al., 2011; Hunt et al., 2011; Fernandez et al., 2013). In addition, on a compositional basis, the complex milk microbiome is notably similar to that of the infant’s oral cavity. Of course, reverse causality might play a role here with milk’s microbiome colonizing the infant’s oral microbiome, and evidence exists to support this (Ruiz et al., 2019; Williams et al., 2019). Aside from retrograde flow of bacteria from the infant’s mouth to the mammary ducts, research also supports the concept that breast skin microbiota may contribute to the milk microbiome, in part because human milk and sebaceous breast skin share a notable number of phylotypes, including *Staphylococcus* species (Latuga et al., 2014). The intriguing presence of anaerobic bacteria (e.g., *Bifidobacteria*) in milk suggests that breast skin and the infant oral cavity may not be the only sources of milk microbiota and suggests the possibility that maternal GI microbiota may also be a source of commensal microbes eventually found in milk. This source of microbes is often referred to as being derived from the entero-mammary route of transfer (Gueimonde et al., 2007; Rodríguez, 2014; Addis et al., 2016). Evidence for the entero-mammary route has been shown in humans, mice, and ruminants (Perez et al., 2007; Treven et al., 2015; Young et al., 2015; de Andrés et al., 2017). Recent findings from animal models imply that a dysfunctional intestinal microbiota can cause mastitis, highlighting the importance of entero-mammary pathways and potential links between mammary and gut health (Ma et al., 2018; Hoque et al., 2022). The myriad ways in which the milk microbiome impacts maternal and infant health are in the early stages of being characterized and understood. Promoting GI health, immune system development, and neural maturation, milk microbiota are thought to aid in the early colonization of the infant GI tract (Jost et al., 2014; Latuga et al., 2014). Our ability to characterize microbial communities has vastly improved with advancements in high-throughput sequencing and omics-based functional profiling technologies (Di Bella et al., 2013). The unique contribution of each omics technique to understanding the milk microbiome, along with the challenges they present, are discussed next.

## Sequencing-based omics approaches

Metagenomics is a powerful tool that has played a key role in the current understanding of the diversity of the human GI microbiome and microbial communities from other anatomical sites (Wang et al., 2015). In addition, metagenomics approaches have been used to successfully discover novel genes and microbial pathways and to identify functional dysbiosis (Hehemann et al., 2010; Qin et al., 2012; Vital et al., 2014), providing a useful template for its application in milk microbiome research. Metagenomes are also helpful for downstream metatranscriptomics and metaproteomics data analysis which depend on metagenomic reference databases. The most prevalent culture-independent approach for characterizing milk microbiota has been 16S rRNA gene amplicon sequencing (Hunt et al., 2011; Pannaraj et al., 2017; Lackey et al., 2019; Moossavi et al., 2019a,b). While this approach can provide information on taxonomic composition, metagenomics can go beyond this application and provide insight into both species and subspecies diversity and functional potential by examining the gene sequences that encode for proteins or functional RNAs (e.g., mRNAs and noncoding RNA). A handful of studies have utilized metagenomic sequencing to characterize the composition of the milk microbiome in bovine (Bhatt et al., 2012; Patel et al., 2017; Kusumawati et al., 2021) and human (Jiménez et al., 2015; Kordy et al., 2020; Seferovic et al., 2020; Olshan et al., 2021) milk but very few have interrogated the functional potential of the microbial communities. Metagenomic deep sequencing was used to compare the microbiomes of milk obtained from cows with clinical mastitis and healthy control cows where functional annotation of the metagenomic sequences was performed to identify differences in metabolic pathways (Hoque et al., 2019). Genes associated with bacterial colonization, proliferation, chemotaxis and invasion, oxidative stress, biofilm formation, and antimicrobial resistance, among others, were overrepresented in bacteria found in milk produced by cows with clinical mastitis (Hoque et al., 2019). Another study carried out metagenomic sequencing of milk produced by cows with subclinical mastitis and found genes associated with multidrug antibiotic resistance; however, no milk produced by healthy cows was included to assess impact of disease on functional gene presence (Bhatt et al., 2012). Milk is a challenging matrix for microbiome characterization using omics due to its relatively low microbial biomass and high ratio of host-to-microbial DNA, which complicates analyzes focusing on the microbiome (Seferovic et al., 2020; Yap et al., 2020; Moossavi et al., 2021). Metagenomics analysis requires a sufficient quantity of high-quality microbial DNA for high coverage (Wang et al., 2015). Contaminating DNA is also an issue for downstream analysis as it is inversely related to the microbial biomass of samples, underscoring the importance of including extraction and sequencing controls (e.g., extraction/PCR negative controls and mock community positive controls) when investigating milk microbial communities (Dahlberg et al., 2019; Moossavi et al., 2021; Pace et al., 2021). In-depth reviews of shotgun metagenomics that cover aspects such as best practices for study design, sample collection and analysis, library preparation, sequencing and metagenome assembly are available (Quince et al., 2017; Knight et al., 2018).

Metagenomic approaches describe the presence of genes and organisms but are unable to provide information on the transcriptional activity of individual microbes or the microbial community at large. Metatranscriptomics can be used to profile the genes expressed by the entire microbial community. Transcriptomic studies often target protein coding RNA [i.e., messenger RNA (mRNA)], but can also profile noncoding RNAs. Multiple studies have profiled the transcriptome of mammary epithelial cells and have been particularly interested in gene expression changes over the course of lactation stages (Bionaz et al., 2012; Lemay et al., 2013; Bhat et al., 2019; Ji et al., 2019; Lin et al., 2019; Martin Carli et al., 2020). Less is known about the milk metatranscriptome, as host RNA has primarily been mined in milk for information about host cell function rather than microbe function (Zorc et al., 2018). This may be due to several obstacles, such as the challenge of differentiating between host and microbial RNA given the low microbial biomass in milk. mRNA is highly unstable and the amount present in a sample can be obscured by the more abundant ribosomal RNA. Although mRNA enrichment is possible, it may cause potential biases (Li et al., 2019). Total RNA metatranscriptomics offers the possibility to gain taxonomic insights (Li et al., 2019; Xue et al., 2020) in addition to functional information. However, it should be noted that the mere presence of a transcript does not guarantee translation into protein and proteomic (or metaproteomic) analysis is required if an accurate characterization of protein expression is desired. Matched metagenomic data can be helpful for metatranscriptome analyzes and tools have been developed to facilitate metatranscriptome assembly (Ye and Tang, 2015). *De novo* assembly of high-quality reads into transcripts using bioinformatic tools designed for microbial communities is an alternative strategy (Leung et al., 2013; Celaj et al., 2014; Aguiar-Pulido et al., 2016; Shakya et al., 2019). Metatranscriptomics analyzes have been useful in the interrogation of other microbiomes, providing an idea of how this tool should be applied in milk research. Comprehensive reviews on the use of and best practices for metatranscriptomics are available (Bashiardes et al., 2016; Zhang et al., 2021). Integrated host and microbial transcriptome profiling of has been conducted to simultaneously characterize host and microbial functional responses and host–microbe interactions in pediatric asthma (Perez-Losada et al., 2015). This was done by separating transcriptomic (host) and metatranscriptomic (microbiota) sequences *in silico* and mapping to human or microbial databases. A similar approach could be used to uncover host–microbe interactions in the context of the milk microbiome in mastitis.

## Mass spectrometry-based omics approaches

Much of the existing literature that aims to characterize the milk microbiome documents taxonomic population shifts under varying host or environmental conditions, but few studies have examined functional consequences of these shifts. Functional redundancy is known to exist in microbial communities across anatomical sites, and it is critical to go beyond taxonomic cataloging and examine the functional roles and phenotypes of microbial communities in milk and their phenotypic effects on the host (Moya and Ferrer, 2016). To better understand how the functional outputs of the milk microbiome impacts maternal and infant health, research must examine the biomolecules that have a closer relationship to phenotype. Mass spectrometry-based technologies, namely metaproteomics, metabolomics, and lipidomics, have enabled functional characterization of microbial communities in a variety of ecosystems.

Proteomics has been used as a tool to examine protein expression and proteolytic activity of microorganisms, providing insights on the function of fermenting microbiota in dairy products like cheese and yogurt (Gagnaire et al., 2004; Manso et al., 2005; Gagnaire et al., 2009). Several proteomic and peptidomic studies have been conducted to examine changes in milk peptides and proteins due to mastitis (Danielsen et al., 2010; Larsen et al., 2010; Chiaradia et al., 2013; Mansor et al., 2013; Zhang et al., 2015; Thomas et al., 2016). These proteomic studies have been useful for the identification of mastitis and general mastitis susceptibility biomarkers but are host focused unlike metaproteomic studies that assess functionality of the microbiome. One exception was Piras and colleagues who used metaproteomics analyzes of raw bovine milk to identify proteins related to antimicrobial resistance (Piras et al., 2020). Metaproteomics can bridge genetic information obtained from metagenomics and metatranscriptomics to the metabolic and phenotypic information contained in the metabolome (Van Den Bossche et al., 2021). Metaproteomic profiling has been used to understand the functional changes in the human gut microbiome during the pathogenesis of colorectal cancer which is known to be associated with taxonomic alterations in intestinal bacteria (Long et al., 2020). Recently, a free-flow isoelectric focusing (FFIEF) electrophoresis method was used to enrich low-abundance bacterial cells from human saliva where host cells are highly abundant (Jiang et al., 2022). This strategy reduced interference by host proteins and enabled deeper coverage of the oral metaproteome, indicating that it may be valuable in milk metaproteomic studies which face similar challenges of low microbial to host cell ratios and highly abundant host proteins such as casein.

Despite the widespread use of proteomics for single organisms, progress in metaproteomics has been slow. A few different factors have been barriers to the wider use of metaproteomics in studying microbial communities. The analysis of complex multispecies samples such as microbial communities often requires sample pre-fractionation to increase detection of peptides, and by association coverage of the metaproteome (Issa Isaac et al., 2019). This can result in significantly increasing overall sample numbers and therefore study costs. Data analysis is still a major challenge in metaproteomics. Peptide sequences, inferred from MS/MS fragmentation patterns, are computationally assigned to proteins. Mapping peptides to proteins and subsequently taxonomic association within a community containing numerous species that all have proteins with similar peptide sequences (i.e., protein sequence homology) does not discriminate the source of the protein (Kleiner et al., 2017). This is done using databases of predicted protein sequences whose quality depends on the availability and annotation of metagenomes (Hettich et al., 2013). While this is much easier for single organisms with sequenced genomes, microbial community data relies on the availability of sample specific metagenomes or high-quality public databases with comprehensive functional and taxonomic annotation. There is a growing emphasis on library-free methods of identifying and quantifying proteins in proteomics research through recent developments like Kaiko, a deep learning model that is trained to build protein databases directly from proteomics data without the aid of metagenomic sequencing (Lee et al., 2022). Library-free protein analysis could be a particularly advantageous method in milk research given the high degree of taxonomic variation in this biological niche and limited availability of existing protein reference databases. Further advancements in proteomics includes functional profiling of the proteome *via* activity-based protein profiling (ABPP). This method uses function-dependent covalent labeling with activity-based probes to pick out which proteins in a sample are active and has been successfully employed in fecal microbiome analysis (Whidbey et al., 2019; Couvillion et al., 2020). This technology could be useful for the analysis of the milk microbiome where low abundance, yet functionally significant microbial or host proteins are overshadowed by an intensely abundant casein and whey background (O’Donnell et al., 2004). Although some enrichment techniques have been previously developed, like 2D chromatography and fractionation, these methods can be costly and time intensive, and ABPP is a promising alternative (Couvillion et al., 2020). Salvato et al. have presented a detailed introduction of the use of metaproteomics to interrogate host-associated microbiomes (Salvato et al., 2021).

Interactions among the environment, host, and microbes are frequently relayed through small molecule metabolites, making metabolomics a key tool to elucidate host-microbial co-metabolism (Nicholson et al., 2005; D’Souza et al., 2018). Additionally, metabolomics can provide information on pathway regulation, signaling processes, and phenotype (Zhang et al., 2012). Metabolites can be introduced to milk through mammary epithelial cell secretions, somatic cell activity, serum, or microbial metabolism (Sundekilde et al., 2013b). The prevalence of certain metabolites in raw milk can influence storage stability, coagulation properties, heat stability, fermentation properties, and flavor (Cadwallader and Singh, 2009; Sundekilde et al., 2013a; Zhu et al., 2021b). Metabolomic approaches have been prevalent in studies that aim to understand the nutritional value of milk, comparing nutritional components between species, humans of varying geographical location, or milk substitutes such as infant formula (Qian et al., 2016; Scano et al., 2016; Gay et al., 2018; Perrone et al., 2019). Much less prevalent are investigations on the metabolome in mastitic milk. Xi et al. (2017) used untargeted liquid chromatography-quadrupole-time of flight mass spectrometry in the MS^E^ mode to identify metabolites that were different in milk from cows that were healthy or had sub-clinical or clinical mastitis. Downregulation of carbohydrate, energy and lipid metabolism and upregulation of amino acid metabolism was observed in clinical mastitis. Untargeted nuclear magnetic resonance spectroscopy (^1^H-NMR) has also been used to characterize the milk metabolome in dairy cows with mastitis. Results suggest that mastitis was associated with alterations in the tricarboxylic acid cycle (TCA cycle) and phenylalanine, tyrosine and tryptophan biosynthesis (Zhu et al., 2021a). Some recent studies have looked at the milk metabolome and microbiome composition in the context of infant health and maternal diet. Shotgun metagenomics and metabolomics were used to identify significant differences in bacterial and viral species/strains and pathways in the breast milk of subjects with celiac disease on a gluten free diet (Olshan et al., 2021). In another study, metabolome and microbiome profiles of milk produced by women across several countries were compared using NMR and 16S rRNA gene sequencing to reveal specific metabolite profile associations with geographical locations (Gomez-Gallego et al., 2018). Correlations between milk metabolite prevalence and relative abundance bacteria were identified, suggesting potential functional relationships between the milk microbiome and metabolites (Gomez-Gallego et al., 2018).

Lipids are an important class of metabolites that could provide useful insights in mastitis research, given that many inflammatory responses are mediated by bioactive lipids (Dhankhar et al., 2016). Beyond resolving mastitis, milk lipids have been shown to have nutritional and immunomodulatory affects in the consumer, making milk microbiome lipidomics relevant to both dairy consumers and breastfeeding infants (Dhankhar et al., 2016; George et al., 2020, 2021; Hewelt-Belka et al., 2020). Despite how informative the milk lipidome can be, few studies have carried out comprehensive characterization of the milk lipidome in the context of mastitis. A recent study by Ceciliani and colleagues demonstrated significant changes in lipid classes such as triacylglycerols and sphingomyelins in milk from dairy cows with subclinical intramammary infection by non-aureus staphylococci (Ceciliani et al., 2021).

Given the challenges associated with small molecule annotation and identification (Chaleckis et al., 2019), user-friendly milk-specific metabolomic databases such as the Milk Composition Database (MCDB) are a valuable resource for researchers analyzing milk metabolomics data (Foroutan et al., 2019). The milk metabolome represents small molecule metabolites that might originate from the host, microbiome, their co-metabolism, and environmental exposures. This presents a unique analytical challenge of distinguishing between metabolites generated by host and microbial metabolism, which is further complicated by a high background of host-generated metabolites. Metabolic networks, which use databases that map metabolites back to enzyme of production, gene sequence, and then organism genomes, can help to untangle metabolite origin (Biggs et al., 2015; Kanehisa et al., 2017). However, these methods are generally only limited to genes and metabolites whose functions, identities, and relationships have already been extensively modeled. Annotation of metabolite origins *via* networks (AMON) is a recently developed analytical tool that uses genomic information to predict if a metabolite originated from a single organism (the host) or a group of organisms (bacteria), allowing for the analysis of complex untargeted metabolomics (Shaffer et al., 2019). These types of bioinformatic tools may be particularly useful when examining the complex milk metabolome. A recent review by Bauermeister et al. (2022) provides an informative overview on mass-spectrometry based metabolomics data and data analysis approaches.

Table 1 lists the references utilizing the omics techniques discussed above for studying the milk microbiome.

**Table 1:** List of milk microbiome related omics references cited in this review.

Biomolecule of interest	Omics approach	Analytical workflow	References
DNA	Marker gene sequencing	16S rRNA amplicon sequencing	Hunt et al., 2011; Pannaraj et al., 2017; Lackey et al., 2019; Moossavi et al., 2019a,b
	Metagenomics	Next generation sequencing	Bhatt et al., 2012; Jiménez et al., 2015; Patel et al., 2017; Hoque et al., 2019; Kordy et al., 2020; Seferovic et al., 2020; Yap et al., 2020; Kusumawati et al., 2021; Olshan et al., 2021
RNA	Metatranscriptomics		--no metatranscriptomic studies found--
Protein	Metaproteomics	LC-IMS-MS/MS	Piras et al., 2020
Metabolite	Metabolomics	UPLC-Q-TOF MS^E^	Xi et al., 2017
		GC–MS	Olshan et al., 2021
		NMR	Gomez-Gallego et al., 2018; Zhu et al., 2021a
Lipid	Lipidomics	LC–MS/MS	George et al., 2020, 2021; Hewelt-Belka et al., 2020; Ceciliani et al., 2021

## Integration of multi-omics data

In a multi-omics approach, a combination of the strategies mentioned in this review to characterize holistic “milk-omics” profiles can be integrated to investigate the complex dynamics of microbial communities and their impact on the host. By studying the interplay of genes, transcripts, proteins, and metabolites, researchers can obtain a more thorough systems-level understanding of microbial community functions, phenotypes, and their influence on host phenotype. Data generated by each omics approach can be informative about different aspects of the microbial community (Heintz-Buschart and Westerhuis, 2022) and integration of complementary omics data can provide a more holistic understanding (Nyholm et al., 2020). Although there is growing interest in integrating omics data, this is not a trivial task given that each ‘meta’-omics approach involves significant resources (financial and computational) and specialized expertise. Furthermore, studies using multiple platforms of analysis tend to generate disparate forms of high-dimensional data with varying amounts of missing values. Efficient data integration methods are necessary for meaningful interpretation of results, and ongoing development of analytical pipelines and software is making this endeavor more feasible (Noecker et al., 2016; Rohart et al., 2017; Jiang et al., 2019; Tarazona et al., 2020).

Multiple taxonomic and omics approaches have been used in tandem to assess previously mentioned factors of interest like lactation stage differences, dairy product quality, and bioactive molecules but have not focused on microbial functions and their impact on host phenotype (Lu et al., 2013, 2015; Morris et al., 2016; Afshari et al., 2021). Correlations between metagenomic compositional profiles and raw milk metabolites revealed potential associations between bacterial genera and metabolite markers of milk as related to feed practices (Bellassi et al., 2021). By focusing on the association of metabolite profiles and shifts in taxonomy representation due to host or environment perturbations rather than metabolic pathway analysis, these studies show correlation but do not confirm causation. A combination of untargeted metabolomics and 16S rRNA gene sequencing, enabled the analysis of correlative taxonomy and metabolite profile changes during sub-clinical *Streptococcus agalactiae* mastitis (GBS) (Tong et al., 2019). Specific bacteria were highly correlated with several metabolites, suggesting possible functional relationships and potential diagnostic biomarkers and pathway analysis suggested that GBS disrupts the TCA cycle in mammary gland cells (Tong et al., 2019).

Integrated multi-omics studies have been more common in the field of GI microbiome research. Longitudinal proteomics, metabolomics, and metagenomics data have been collected to examine microbiome composition and function (Gierse et al., 2020). Researchers found stability in the proteome and metabolome profiles of fecal samples despite fluctuations in microbe taxonomy, presenting an interesting story of functional redundancy that could not have been told through a single platform of analysis (Gierse et al., 2020). An integrated metagenomic, metatranscriptomic and metaproteomic approach was used to link microbial functional signatures to metabolic traits in distinct taxa in the fecal microbiome for type 1 diabetes mellitus (Heintz-Buschart et al., 2016). Similar longitudinal multi-omic approaches could prove insightful in milk microbiome research.

## Conclusions and future directions

In this review we draw attention to a relative lack of research on relationships among the milk microbial community, its functional capacity, and ultimate impact on the proteome, lipidome and metabolome of milk – collectively referred to here as milk-omics. To more completely understand how the milk microbiome impacts maternal and infant health, research should incorporate multi-omics approaches that profile the analytes that have a closer relationship to phenotype, such as RNA, proteins, and metabolites. Although several omics studies have been conducted in milk, most have focused on molecules that are produced by the host. Despite the use of multi-omic approaches to study host-associated microbial communities in other anatomical sites, investigations of the milk microbiome have largely been limited to metagenomics and metabolomics. Uncovering the changes in microbial functional output could be a useful next step in understanding the development of mastitis. Future research must also encompass non-bacterial microbial community members such as fungi, viruses, archaea, and protists and their role in host health and disease. Mastitis that requires clinical intervention is generally treated with antibiotics, but recent studies conducted in other sites of dysbiosis, like the GI tract and vagina, suggest that it may be more beneficial to foster the colonization of health promoting bacteria as opposed to eliminating pathogenic bacteria through antibiotics (Spencer, 2008; Schmidt et al., 2020; Chen et al., 2021). Milk-omics research would also benefit from standardization of methods for recording metadata, sample collection/storage, analysis and data curation (Eloe-Fadrosh et al., 2021). This would increase the generalizability of findings across studies. Inter-individual variations in milk composition points to the need for factoring in metadata regarding maternal diet, genotype, environment, and birth mode to be able to make meaningful comparisons of findings between individuals, cohorts, or studies.

The field of milk microbiome research is an actively developing area of research, and the question of who is doing what in this ecological niche is still largely unexplored. As further investigations are conducted, we advocate for employing integrated multi-omics approaches that go beyond compositional profiling in order to gain a comprehensive mechanistic understanding for the impact of the milk microbiome on the lactating mother and the nursing infant or consumer of dairy products. There is an overarching need for standardization of methods, from sample and metadata collection to data integration and interpretation. The development and adoption of community-wide metadata standards and data generation pipelines, open-source bioinformatic tools and workflows and also making data FAIR (Wilkinson et al., 2016) will empower the milk microbiome research community and accelerate discovery and innovation.

## Funding

This work was supported by the National Institutes of Health NICHD R01 HD092297, US Department of Agriculture, and the USDA National Institute of Food and Agriculture, Hatch project IDA01643.

## Author contributions

SC and TM initiated and conceptualized the review topic. KM and SC worked on the initial draft. All authors contributed to the literature review, manuscript writing, and approved the submitted version.

## Conflict of interest

The authors declare that the research was conducted in the absence of any commercial or financial relationships that could be construed as a potential conflict of interest.

## Publisher’s note

All claims expressed in this article are solely those of the authors and do not necessarily represent those of their affiliated organizations, or those of the publisher, the editors and the reviewers. Any product that may be evaluated in this article, or claim that may be made by its manufacturer, is not guaranteed or endorsed by the publisher.
